# Augmented repair of radial meniscus tear with biomimetic electrospun scaffold: an in vitro mechanical analysis

**DOI:** 10.1186/s40634-016-0058-0

**Published:** 2016-09-13

**Authors:** Benjamin B. Rothrauff, Piya-on Numpaisal, Brian B. Lauro, Peter G. Alexander, Richard E. Debski, Volker Musahl, Rocky S. Tuan

**Affiliations:** 1 Center for Cellular and Molecular Engineering, Department of Orthopaedic Surgery, University of Pittsburgh School of Medicine, 450 Technology Drive, Room 221, Pittsburgh, PA 15219 USA; 2 Orthopaedic Robotics Laboratory, Department of Orthopaedic Surgery, University of Pittsburgh, 300 Technology Drive, Pittsburgh, PA USA; 3 Department of Bioengineering, Swanson School of Engineering, University of Pittsburgh, Pittsburgh, PA USA; 4 McGowan Institute for Regenerative Medicine, University of Pittsburgh, Pittsburgh, PA 15219 USA; 5 College of Medicine, National Taiwan University, Taipei, Taiwan

**Keywords:** Meniscus repair, Radial tear, Scaffold

## Abstract

**Background:**

Large radial tears that disrupt the circumferential fibers of the meniscus are associated with reduced meniscal function and increased risk of joint degeneration. Electrospun fibrous scaffolds can mimic the topography and mechanics of fibrocartilaginous tissues and simultaneously serve as carriers of cells and growth factors, yet their incorporation into clinically relevant suture repair techniques for radial meniscus tears is unexplored. The purposes of this study were to (1) evaluate the effect of fiber orientation on the tensile properties and suture-retention strength of multilayered electrospun scaffolds and (2) determine the mechanical effects of scaffold inclusion within a surgical repair of a simulated radial meniscal tear. The experimental hypothesis was that augmentation with a multilayered scaffold would not compromise the strength of the repair.

**Methods:**

Three multilayered electrospun scaffolds with different fiber orientations were fabricated–aligned, random, and biomimetic. The biomimetic scaffold was comprised of four layers in the following order (deep to superficial)–aligned longitudinal, aligned transverse, aligned longitudinal, and random–respectively corresponding to circumferential, radial, circumferential, and superficial collagen fibers of the native meniscus. Material properties (i.e., ultimate stress, modulus, etc.) of the scaffolds were determined in the parallel and perpendicular directions, as was suture retention strength. Complete radial tears of lateral bovine meniscus explants were repaired with a double horizontal mattress suture technique, with or without inclusion of the biomimetic scaffold sheath. Both repair groups, as well as native controls, were cyclically loaded between 5 and 20 N for 500 cycles and then loaded to failure. Clamp-to-clamp distance (i.e., residual elongation) was measured following various cycles. Ultimate load, ultimate elongation, and stiffness, were also determined. Group differences were evaluated by one-way ANOVA or Student’s *t*-test where appropriate.

**Results:**

Aligned scaffolds possessed the most anisotropic mechanical properties, whereas random scaffolds showed uniform properties in the parallel and perpendicular directions. In comparison, the biomimetic scaffold possessed moduli in the parallel (68.7 ± 14.7 MPa) and perpendicular (39.4 ± 11.6 MPa) directions that respectively approximate the reported circumferential and radial tensile properties of native menisci. The ultimate suture retention load of the biomimetic scaffold in the parallel direction (7.2 ± 1.6 N) was significantly higher than all other conditions (*p* < 0.001). Biomimetic scaffold augmentation did not compromise mechanical properties when compared against suture repair in terms of residual elongation after 500 cycles (scaffold: 5.05 ± 0.89 mm vs. repair: 4.78 ± 1.24 mm), ultimate failure load (137.1 ± 31.0 N vs. 124.4 ± 21.4 N), ultimate elongation (12.09 ± 5.89 mm vs. 10.14 ± 4.61 mm), and stiffness (20.8 ± 3.6 vs. 18.4 ± 4.7 N/mm).

**Conclusions:**

While multilayered scaffold sheets were successfully fabricated to mimic the ultrastructure and anisotropic tensile properties of native menisci, improvements in suture retention strength or adoption of superior surgical techniques will be needed to further enhance the mechanical strength of repairs of radial meniscal tears.

## Background

Meniscus tears involving the central region remain a formidable challenge to orthopaedic surgeons, as the absence of vasculature and a complex loading environment prevent a robust healing response (Abrams et al. 2013; Arnoczky and Warren 1983; Fox et al. 2015). Compared with vertical tears, radial/flap tears, in which there can be disruption of the circumferential fibers, are associated with articular cartilage lesions of increasing severity (Henry et al. 2012). The standard treatment of partial meniscectomy often alleviates pain and mechanical symptoms in the short term but is known to accelerate joint degeneration by increasing contact stresses (Fairbank 1948; Ode et al. 2012). Recent in vitro studies have demonstrated that contact stresses do not differ from native controls until a full-thickness radial tear exceeds 90 % of the meniscus width, prompting renewed efforts to preserve meniscus structure through primary suture repair (Bedi et al. 2012; Mononen et al. 2013; Muriuki et al. 2011; Ode et al. 2012).

Clinical studies have reported variable healing rates of repaired meniscal tears involving the avascular region, attributable to differences in tear morphology, tissue quality, and surgical technique (Choi et al. 2010; Henning et al. 1991; Rubman et al. 1998). However, it remains unknown whether successful healing, defined most commonly by neotissue formation as observed by arthroscopy or MRI, restores native meniscus structure and function, thereby maintaining its chondroprotective role in the articular joint. In a canine model, Newman et al. (1989) found that “healed” radial tear defects consisted of a 3–5 mm gap of fibrovascular scar that failed to restore normal tissue mechanics. As a result, numerous surgical techniques have been explored in an effort to improve repair strength and maintain apposition of the torn edges (Beamer et al. 2015; Branch et al. 2015; Herbort et al. 2010; Lee et al. 2012; Matsubara et al. 2012). Similarly, though largely unexplored in regards to meniscal tears, scaffold sheets can minimize gap formation and augment mechanical properties of surgical repairs of musculoskeletal tissues (McCarron et al. 2010, 2012). To provide mechanical support, scaffolds must possess material properties equivalent to the native tissue, while biological support through the delivery of cells or biological agents must at minimum not compromise the integrity of the surgical repair (Aurora et al. 2012).

To that end, tissue engineering strategies including the independent or combinatorial use of cells, scaffolds, and growth factors, have been increasingly investigated as a means of enhancing the healing response (Moran et al. 2015; Yu et al. 2015). In particular, electrospun nanofibers composed of biodegradable polymers can mimic the topographical and mechanical cues of dense fibrocartilaginous tissues, driving fibrochondrogenic differentiation of seeded mesenchymal stem cells (MSCs) (Baker et al. 2010) and enhancing neotissue formation when placed within a vertical tear in vivo (Baker et al. 2012; Qu et al. 2015). Fisher et al. (2015) recently fabricated a multilayered cell-seeded nanofibrous scaffold capable of mimicking the anisotropic tensile properties of the meniscus that are derived from the complex organization of circumferential and radial tie fibers (Fox et al. 2015). Similarly, our recent report showed that a cell-seeded electrospun nanofibrous scaffold enhanced the mechanical and histological properties of an in vitro repair model of a radial meniscus tear (Shimomura et al. 2015).

Because the mechanical effect of incorporating an electrospun scaffold that structurally mimics meniscus fibrous architecture within a surgical repair is unknown, the purposes of this study were to (1) evaluate the effect of fiber orientation on the tensile properties and suture-retention strength of multilayered electrospun scaffolds and (2) determine the mechanical effects of scaffold inclusion within a surgical repair of a simulated radial meniscal tear. The experimental hypothesis was that augmentation with a multilayered scaffold would not compromise the strength of the repair.

## Methods

### Study design

Individual sheets of aligned or randomly oriented electrospun nano/microfibers were combined to form biomimetic multilayered scaffolds. In addition to fiber diameter characterization, the tensile and suture-retention properties of the scaffolds were determined. The biomimetic scaffold, modeling the fibrous structure of the native meniscus, was incorporated as a sheath enveloping the tibia and femoral surfaces of a radially transected lateral bovine meniscus. The mechanical properties of the suture repair, with or without inclusion of the biomimetic scaffold, were determined following cyclic loading and subsequent load to failure.

### Fabrication of multilayered nanofibrous scaffold

**Fig. 1:**
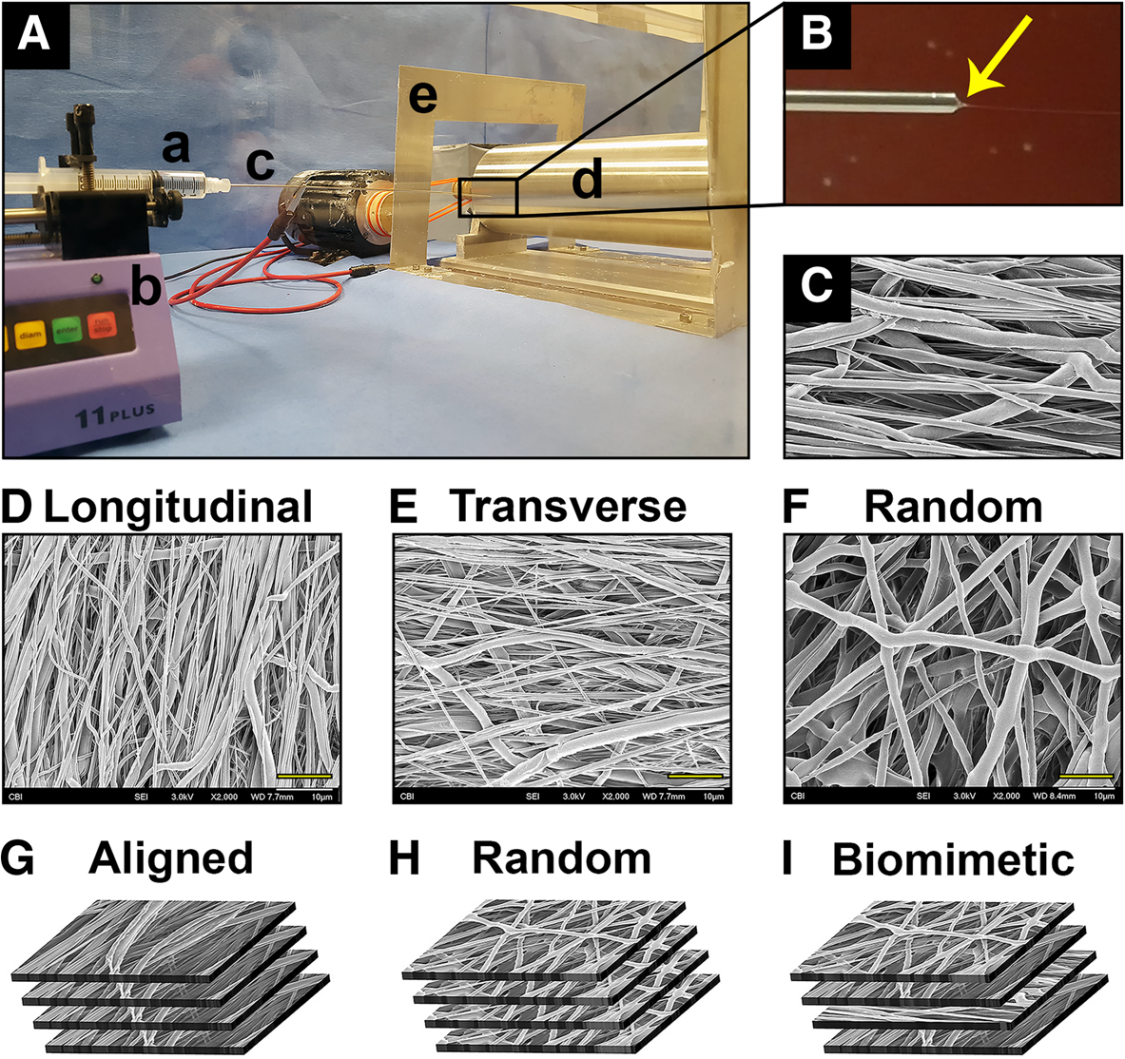
Fabrication of multilayered electrospun scaffolds. (**A**) Electrospinning apparatus consisting of (**a**) syringe with polymer solution, (**b**) syringe pump, (**c**) 18-gauge blunt tip needle, (**d**) rotating mandrel, and (**e**) aluminum shield. (**B**) Taylor cone (*arrow*) with emerging polymer fiber creates (**C**) nanofibrous sheet. (**D**–**F**) SEM images of fiber orientations comprising individual layers. Scale bar, 10 μm. (**G**–**I**) Individual layers are combined to form three types of multilayered scaffolds, (**G**) aligned, (**H**) random, and (**I**) biomimetic (consisting of alternating layers of aligned and random layers)

**Fig. 2:**
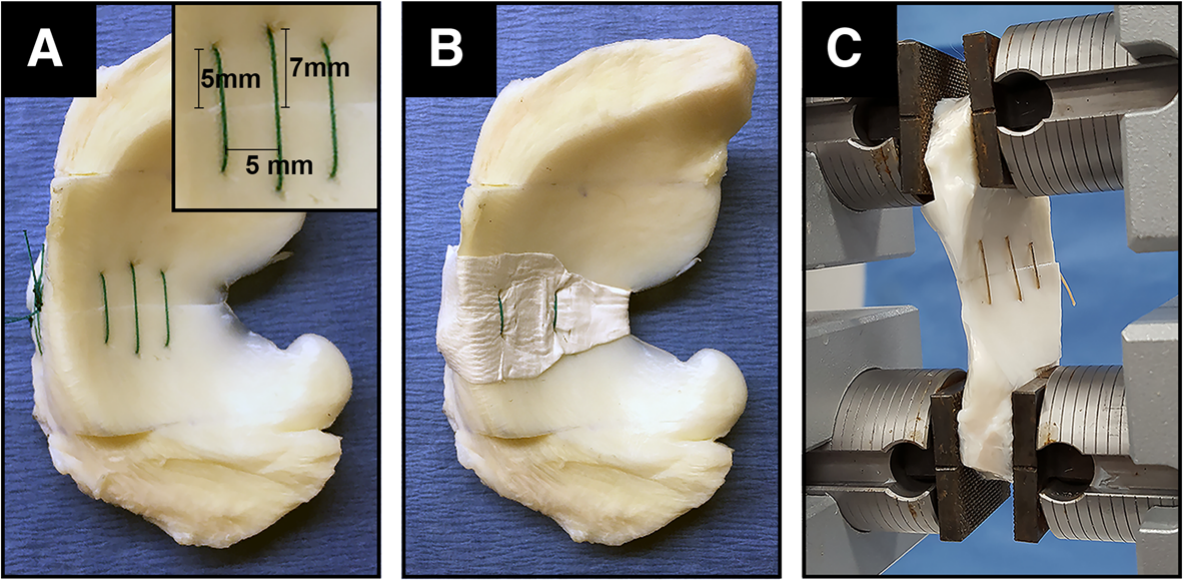
Suture repair of meniscal tears and mechanical testing set-up. **a** Suture repair of fully transected meniscus. Inset shows dimensions of suture placement. **b** Scaffold-augmented repair. **c** Suture repaired meniscus clamped in materials testing machine prior to tensile loading protocol

**Fig. 3:**
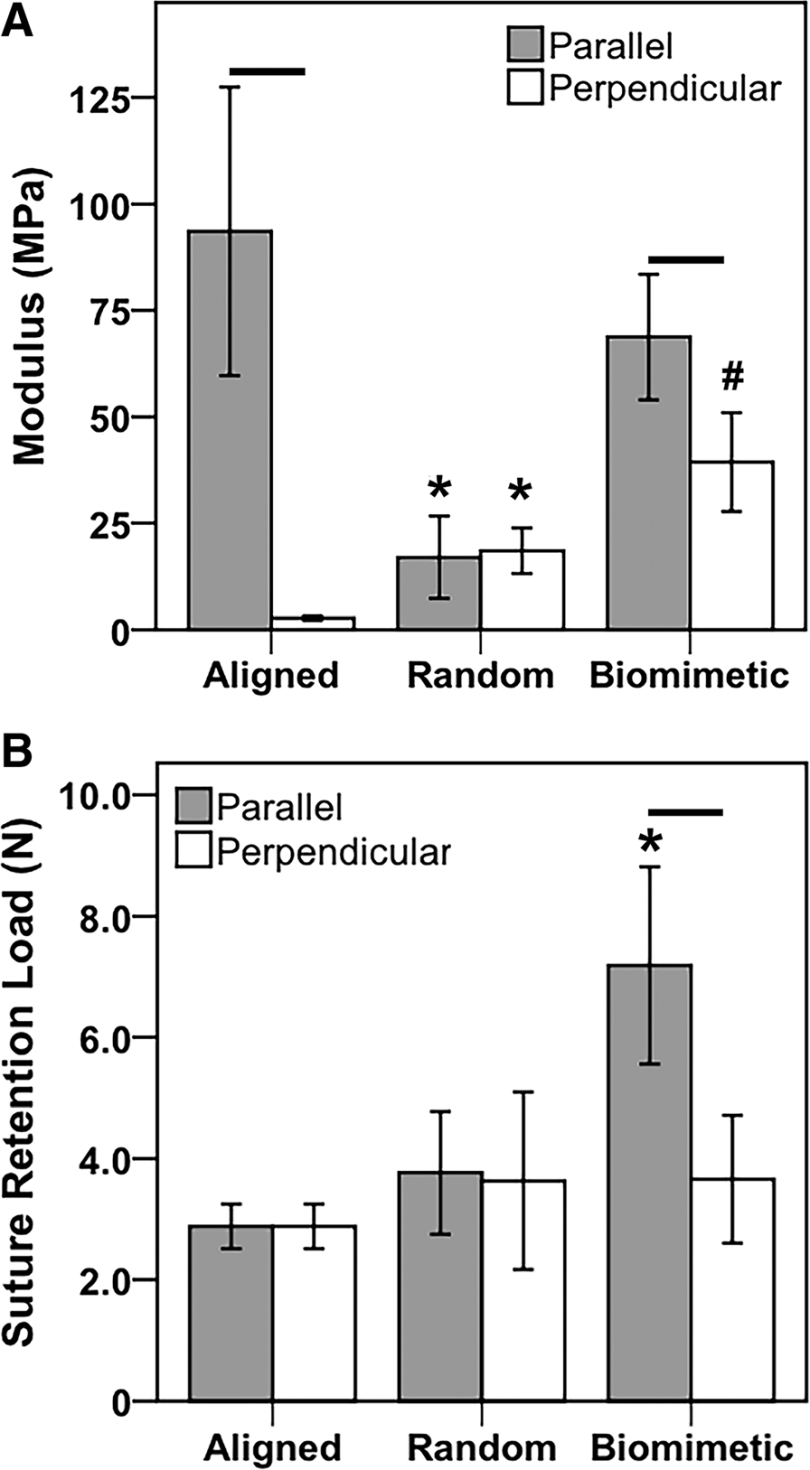
Moduli and suture retention strength of multilayered scaffolds. **a** Tensile modulus of three scaffold designs in parallel (i.e., circumferential) and perpendicular (i.e., radial) direction. **b** Ultimate suture retention load by scaffold design. * (*p* <0.05) and # (*p* <0.001) indicate significant difference across scaffold types for a given direction. Horizontal lines above columns indicate a significant difference (*p* < 0.001) between directions for a given scaffold type

Multilayered nanofibrous scaffolds were fabricated through electrospinning, as shown in Fig. 1. The electrospinning apparatus is shown in Fig. 1A–C. Three orientations of fibers–aligned longitudinal (Fig. 1D), aligned transverse (Fig. 1E), and random (Fig. 1F)–were utilized to create three designs of multilayered scaffolds–(1) aligned (Fig. 1G), (2) random (Fig. 1H), or (3) biomimetic (Fig. 1I). The biomimetic scaffold was comprised of four layers in the following order (deep to superficial): aligned longitudinal, aligned transverse, aligned longitudinal, and random. This design was inspired by the fibrous structure of the native meniscus, in which the circumferential collagen fibers resist hoop stresses (aligned longitudinal) while the tie fibers resist radial stresses (aligned transverse) and random fibers constitute the meniscal surfaces (random) (Fox et al. 2015; Makris et al. 2011). Each layer was fabricated from a solution of poly-ε-caprolactone (PCL, MW = 70 k-90 kd, Sigma-Aldrich, St. Louis, MO) prepared at 15 % w/v in 1:1 (v/v) tetrahydrofuran (THF, Sigma-Aldrich):dimethylformamide (DMF, Sigma-Aldrich). The PCL solution was loaded into a 10 ml syringe and extruded through an 18-gauge blunt tip needle at 3.0 mL/h using a syringe pump (PY2 70-2209; Harvard Apparatus, Holliston, MA). The needle tip was placed 10 cm from a custom-designed cylindrical mandrel, which rotated at a surface velocity of 10 m/s for aligned fibers or 0.5 m/s for random fibers. 10–18 kV DC potential (Gamma High Voltage, Ormand Beach, FL) was applied to the polymer solution while an 8 kV potential was applied to two aluminum shields placed perpendicular to the mandrel axis but parallel to the needle axis (Fig. 1A). For the biomimetic scaffolds, a given layer was removed, reoriented, and reattached to the mandrel such that the fibers of the subsequent layer were electrospun directly onto the former.

### Characterization of multilayered nanofibrous scaffold

Average fiber diameter in each layer was determined by scanning electron microscopy (SEM). Briefly, scaffold layers were dried under vacuum and mounted on aluminum stubs, sputter-coated with 4.5 nm of gold, and imaged by SEM (field emission, JEOL JSM6335F, Peabody, MA) operated at 3 kV accelerating voltage and 8 mm working distance. Images were morphometrically analyzed using Image J (National Institutes of Health, Bethesda, MD).

Each multilayered scaffold was cut into dumbbell-shaped constructs with a central rectangular area measuring 25 mm by 5 mm. Construct thickness was measured with digital calipers at three sites and averaged, from which the cross-sectional area (CSA) was calculated. Constructs were then clamped into a materials testing machine (Model 4502; Instron, Norwood, MA) and loaded under tension in a direction either parallel or perpendicular to fiber alignment. This distinction was arbitrary for random scaffolds. For the biomimetic scaffold, the scaffold was oriented with the two aligned longitudinal layers defining parallel. After preloading to 0.5 N, constructs were preconditioned from 0 to 2 % strain (estimated from clamp-to-clamp distance) for 15 cycles at 20 mm/min before undergoing load to failure at the same elongation rate. A custom digital motion tracking system (Spica Technology, Kihei, Maui, HI; 0.01 mm accuracy) was used to track the vertical displacement of the strain markers (black pen) using a single video camera aligned perpendicular to the plane. These data were inputted into ABAQUS software (ABAQUS/CAE Student Version 6.4; Simulia, Providence, RI) to determine strain. Both structural and material properties were determined. Of note, stiffness (modulus) was determined from the slope of the linear region of the load-elongation (stress-strain) curve while yield load (stress) and yield elongation (strain) were found at the intersection of the data curve and the tangent line with a 0.2 % positive offset along the x-axis, as described previously (Czaplewski et al. 2014).

To determine suture retention strength, scaffolds were clamped on both ends, as in the tensile testing protocol described above, and evenly transected. A single loop of 3-0 prolene suture was passed through the midline of each construct at a distance of 5 mm from the cut edge and secured to a immovable cylindrical rod mounted on the material testing machine. A preload of 0.5 N was applied before loading to failure at 20 mm/min. The maximum load was recorded as the suture retention strength.

### Suture repair of radial tear of lateral meniscus

Twenty-four fresh-frozen lateral menisci of adult cows (2–3 years old, JW Treuth & Sons Inc., Catonsville, MD) were used to simulate repair of a radial tear. Menisci were radially transected in the midbody, beginning in the central region and extending to a width of 90 %, corresponding to a length of ~27 mm (out of 30 mm). A single horizontal stitch of 2–0 braided polyester suture (TiCron, Covidien, Dublin, Ireland) was placed 7 mm from the tear edges and in the center of the tear width to reduce the transected edges (Fig. 2a). The remaining 10 % width was then transected to complete the tear. In the scaffold-augmented group, an hour-glass shaped biomimetic scaffold was wrapped around the tear site so as to cover the femoral and tibial surfaces of the meniscus. The scaffold was oriented such that the two layers of aligned longitudinal nanofibers were parallel to the circumferential fibers of the meniscus. For both the suture repair and scaffold-augmented groups (*n* = 8 per group), a double horizontal mattress suture technique was subsequently performed in which the sutures were positioned 5 mm medial and lateral to the reducing suture and 5 mm from the tear margin (Fig. 2a, b). The double horizontal sutures passed through, and laid superficial to, the scaffold sheath so as to ensure its stable incorporation into the repair. Native menisci served as intact controls against which both repair groups were compared.

### Mechanical testing of suture repair

Three groups of menisci were tested–(1) intact controls, (2) suture repairs, and (3) scaffold-augmented repairs, as described above. For each, the anterior and posterior portions of the menisci were trimmed to provide relatively flat surfaces for clamping (Fig. 2). Menisci were clamped in a materials testing machine such that the axis of tension was perpendicular to the simulated tear (Fig. 2c). After preloading to 5 N, the construct was cyclically loaded from 5 to 20 N for 500 cycles at a rate of 20 mm/min, pausing after 250 cycles to tighten the clamps. Thereafter, the construct was loaded to failure at the same rate. Residual elongation (indicative of gap formation) was determined after 1, 10, 50, 100, 250, and 500 cycles. Ultimate load, ultimate elongation, and stiffness were determined from the load to failure.

### Statistical analysis

A priori power calculations utilizing pilot data and values from relevant literature revealed a minimum sample size of seven was required to detect a 15 % difference in ultimate load when comparing scaffold-augmented repairs against suture repair alone (power = 0.80, *α* = 0.05). As a result, eight menisci were allocated to each group for mechanical testing. In analyzing scaffold properties, a two-way ANOVA with fixed factors–scaffold type (3) and direction (2)–was employed to determine main and interactive effects. Subsequent one-way ANOVA with Tukey post-hoc tests or Student’s *t*-tests were performed to determine statistical differences between conditions. One-way ANOVA with post-hoc Tukey’s test was performed to evaluate differences in native meniscus, suture repairs, and scaffold-augmented repairs, after undergoing both cyclic loading and load to failure. *p* < 0.05 was considered statistically significant.

## Results

### Scaffold properties

Three orientations of PCL fibers–(1) aligned longitudinal (fiber diameter: 811 ± 388 nm, Fig. 1D), (2) aligned transverse (diameter: 772 ± 408 nm, Fig. 1E), and (3) random (diameter: 1562 ± 524 nm, Fig. 1F)–were successfully prepared by electrospinning. From these orientations, three multilayered scaffolds were fabricated–(1) aligned (thickness: 0.60 ± 0.04 mm, Fig. 1G), (2) random (thickness: 0.37 ± 0.08 mm, Fig. 1H), and (3) biomimetic (thickness: 0.56 ± 0.05 mm, Fig. 1I). Both structural and material tensile properties were determined, with the latter presented in Table 1. For all parameters tested, the aligned scaffolds possessed the greatest degree of anisotropy, i.e., a difference when comparing values in the parallel and perpendicular direction (*p* < 0.001). Conversely, random scaffolds demonstrated isotropic properties, with no significant differences between the two directions. The biomimetic scaffold, much like the native meniscus, exhibited higher values for ultimate stress (*p* < 0.001), yield stress (*p* = 0.002), and modulus (*p* < 0.001, Fig. 3a), in the parallel (i.e., circumferential) direction, as compared to the perpendicular (i.e., radial). The biomimetic scaffold possessed lower material properties than the aligned scaffolds in the parallel direction, although these differences did not consistently reach statistical significance. Conversely, the biomimetic scaffold was superior to both the aligned and random scaffolds when considering the perpendicular direction, as found for ultimate stress (*p* < 0.001 vs. aligned, *p* = 0.042 vs. random), yield stress (*p* < 0.001 vs. aligned, *p* = 0.046 vs. random), and modulus (*p* < 0.001 vs. both). Nevertheless, no scaffold, regardless of direction, possessed a high resistance to suture pull-through (Fig. 3b), even though the biomimetic scaffold was significantly stronger than either scaffold when oriented in the parallel direction (*p* < 0.001). This finding, coupled with its anisotropic material properties reminiscent of native menisci, supported the surgical incorporation of the biomimetic scaffold positioned such that the two layers of aligned longitudinal fibers (i.e., parallel in Table 1) were parallel to the circumferential fibers of the underlying meniscus (Fig. 2b).

**Table 1:** Material Properties of Scaffold Designs^a^

	Aligned	Random	Biomimetic
Ultimate Stress (MPa)			
Parallel	12.9 ± 4.3^*, ***^	3.4 ± 1.1^***^	8.5 ± 1.9^*, ***^
Perpendicular	1.2 ± 0.3^****^	3.8 ± 1.0^***^	5.1 ± 1.0^***^
Ultimate Strain (mm/mm)			
Parallel	0.40 ± 0.03^*^	3.28 ± 1.49^****^	0.34 ± 0.08
Perpendicular	3.69 ± 1.30	3.00 ± 1.46	0.40 ± 0.07^***^
Modulus (MPa)			
Parallel	93.6 ± 33.9^*, *****^	16.9 ± 9.7	68.7 ± 14.7^*, *****^
Perpendicular	2.7 ± 0.5^***^	18.5 ± 5.4^***^	39.4 ± 11.6^****^
Yield Stress (MPa)			
Parallel	4.9 ± 1.5^*, *****^	1.7 ± 0.5	4.0 ± 1.3^**, ******^
Perpendicular	0.4 ± 0.1^****^	1.7 ± 0.4^***^	2.2 ± 0.4^***^
Yield Strain (mm/mm)			
Parallel	0.06 ± 0.01^*****^	0.11 ± 0.04	0.07 ± 0.01^*****^
Perpendicular	0.12 ± 0.07	0.10 ± 0.04	0.07 ± 0.02

^a^For a given scaffold type, significant difference when comparing parallel vs. perpendicular direction, ^*^ *p* < 0.001, ^**^ *p* < 0.05; Significantly different from both scaffolds, ^***^ *p* < 0.05, ^****^ *p* < 0.001; Significantly different from random scaffold, ^*****^ *p* < 0.01, ^******^ *p* < 0.05

### Meniscus repair properties

Residual elongation values following cyclic loading are shown in Table 2. All repairs survived the cyclic loading protocol and exhibited significantly larger displacement at a given cycle when compared against native controls (*p* < 0.001). There was no statistical difference between the repair groups. Similarly, both repair groups demonstrated significantly lower ultimate failure load (*p* < 0.001) and stiffness (*p* < 0.001) than the native control, yet no differences between repair groups were found (Table 3). All repaired constructs failed by suture breakage, whereas the native meniscus specimens experienced clamp slippage.

**Table 2:** Residual Elongation (mm) During 500 Cycles Between 5 and 20 N

Cycle	Native	Suture Repair	Scaffold-Augmented
1	0.26 ± 0.16^a^	1.14 ± 0.28	1.27 ± 0.38
10	0.40 ± 0.23^a^	1.75 ± 0.40	1.99 ± 0.33
50	0.55 ± 0.33^a^	2.57 ± 0.57	2.93 ± 0.35
100	0.66 ± 0.39^a^	3.15 ± 0.75	3.58 ± 0.47
250	0.86 ± 0.51^a^	4.29 ± 1.17	4.88 ± 0.80
500	0.93 ± 0.49^a^	4.78 ± 1.24	5.05 ± 0.89

^a^Native control significantly less (*p* < 0.001) than either repair group at given cycle

**Table 3:** Mechanical Properties of Native and Repaired Menisci Pulled to Failure

	Native	Suture Repair	Scaffold-Augmented
Ultimate Load (N)^a^	437.3 ± 117.5	124.4 ± 21.4	137.1 ± 31.0
Ultimate Elongation (mm)^b^	5.12 ± 1.55	10.14 ± 4.61	12.09 ± 5.89
Stiffness (N/mm)	141.0 ± 42.4^a^	18.4 ± 4.7	20.8 ± 3.6

^a^Native control significantly greater (*p* < 0.001) than either repair group

^b^Scaffold-augmented group significantly greater (*p* = 0.022) than native control

## Discussion

In this study, an electrospun scaffold that mimicked the fibrous architecture and anisotropic mechanical properties of the meniscus was fabricated. The biomimetic scaffold was incorporated within a double horizontal mattress suture repair of a complete radial tear without diminishing the mechanical properties of the repair. The biomimetic scaffold contained two layers of aligned nanofibers, imparting the greatest tensile strength in a direction parallel to these fibers, while a layer of aligned fibers oriented transversely resisted tension applied in the perpendicular direction. When used as an augmentation to meniscal repair, these fibers are intended to mimic the circumferential and radial tie fibers of the native meniscus, respectively (Fithian et al. 1990). Similarly, the layer of randomly oriented fibers mimics the surface of the native meniscus (Fithian et al. 1990; Fox et al. 2015). The anisotropic tensile properties of the biomimetic scaffold grossly matched those of native meniscus as well. Namely, the average modulus of the scaffold in the parallel (i.e. circumferential) direction was 67.8 ± 14.7 MPa, which falls within the range of reported modulus values (~59 to 294 MPa) of circumferentially oriented specimens obtained from both bovine and human menisci (Fithian et al. 1990; Lechner et al. 2000; Proctor et al. 1989; Tissakht and Ahmed 1995). Similarly, the scaffold modulus in the perpendicular direction (39.4 ± 11.6 MPa) was within the range of native meniscus specimens (~3 to 60 MPa) (Fithian et al. 1990; Tissakht and Ahmed 1995). As the range of values suggests, the material properties of native menisci vary broadly by region (Fithian et al. 1990; Proctor et al. 1989; Tissakht and Ahmed 1995) and are further affected by the cross-sectional area of the specimen undergoing tensile testing (Lechner et al. 2000). The scaffold fabrication parameters utilized in this study could be modified to provide increased stiffness in the circumferential direction with corresponding reductions in the radial direction, as exaggerated in the aligned scaffold. The resulting moduli would then more closely match the average reported moduli of native menisci. However, the superficial regions of the menisci exhibit more isotropic properties than the deeper regions, a finding worthy of consideration when implementing a scaffold as a sheath, such as in this study (Fithian et al. 1990).

While the biomimetic scaffold mimicked the topography and tensile properties of native menisci, it did not improve the mechanical properties of a simulated radial meniscal tear repaired with suture. Both the ultimate failure load (~125 N) and stiffness (~19 N/mm) of the repair and scaffold-augmented groups are comparable to reported values using similar suture techniques in cadaveric models (Beamer et al. 2015; Bhatia et al. 2015; Branch et al. 2015; Herbort et al. 2010). In testing different suture techniques, Herbort et al. (2010) and Branch et al. (2015) confirmed that multiple sutures are superior to a single suture loop, providing in vitro support to the clinical standard of using an inside-out double horizontal suture technique for repair of radial tears. In this study, a single horizontal stitch was placed equidistant from the central and peripheral rims to reduce the torn edges before performing a double horizontal mattress suture repair, with or without inclusion of the biomimetic scaffold serving as a sheath. As visualization of tear apposition was impossible with the opaque scaffold in place, the reducing stitch was necessary. This third stitch likely accounts for the elevated ultimate load found in this study, as compared against those reported by Herbort et al. (2010) (109 N) and Bhatia et al. (2015) (106 N) when a double horizontal suture technique was evaluated. Unfortunately, the scaffold also prevented direct visualization of markers that might otherwise be used to track tissue strain and gap formation, as utilized in related studies (Beamer et al. 2015; Bhatia et al. 2015). Consequently, residual elongation (i.e. clamp-to-clamp distance) was used to indicate gap formation, although the ~1 mm elongation found after 500 cycles in the native controls suggests that clamp slippage and/or viscoelastic creep partially contributed to this measurement of gap formation.

The biomimetic scaffold of this study enveloped the tear site and was secured as part of the double horizontal suture repair. This approach, inspired by the application of scaffold sheets to augment rotator cuff repairs (Ratcliffe et al. 2015), stands in contrast to a previous clinical study in which autologous fascia was used to wrap surgical repairs of complex meniscal tears (Henning et al. 1991). Namely, the fascia sheath was not intended to provide any mechanical support to the suture repair and therefore was attached to the meniscus only along the peripheral rim. In this study, suture breakage was the mode of failure for all repairs, obviating any possible benefit of including a mechanically robust scaffold. If improvements in surgical materials or techniques were sufficient to alter the mechanism of failure to suture pull-through (as seen with rotator cuff repairs), augmentation with mechanically robust scaffolds could further enhance repair strength.

In order to provide mechanical support to the surgical repair, the scaffold should possess (1) material properties similar to that of the native tissue and (2) suture retention strength equal to, or greater than, the surgical repair (Aurora et al. 2012). While the biomimetic scaffold met the former criterion, it exhibited a poor ability to hold suture with an ultimate pullout load of 7.2 ± 1.6 N. Categorically, nonwoven electrospun nanofibers do not adequately hold suture. However, the implementation of textile patterns such as braiding or weaving could provide added resistance to suture pull-through (Hakimi et al. 2015; McCarron et al. 2010). To that end, future studies will explore fabrication methods capable of enhancing the suture retention strength of the biomimetic scaffold. Similarly, in vitro culture of cells on the biomimetic scaffold may increase mechanical properties through deposition of extracellular matrix proteins (Fisher et al. 2015) while also contributing to neotissue formation if localized to the lesion (Shimomura et al. 2015).

Baek et al. (2015) and Fisher et al. (2015) independently developed cell-seeded multilayered electrospun scaffolds that mimicked the anisotropic fibrous ultrastructure of the meniscus. When implanted within a vertical longitudinal tear created within an explant (Baek et al. 2016) and animal (Qu et al. 2015) model, these electrospun scaffolds promoted neotissue formation within the tear site. In contrast, the biomimetic scaffold of the present study was applied as a sheath enveloping the tear site. Application of the scaffold as a sheath is not biomimetic in the same sense as the former electrospun scaffolds placed within the body of the meniscus, whereby through the process of contact guidance the scaffolds may direct cells to deposit collagen fibers in the same orientation as the circumferential and radial fibers of the surrounding native tissue (Baek et al. 2016; Baker et al. 2010). On the other hand, radial tears are less amenable to the stable localization of scaffolds within the tear site as compared to longitudinal or horizontal tears, given the greater propensity for gapping when the meniscus is loaded. Therefore, the application of the scaffold as a sheath, if drawing lessons from augmented rotator cuff repairs (Aurora et al. 2012), could theoretically off-load the surgical repair while also protecting a cell-seeded construct placed within the defect site. Furthermore, we recently demonstrated in an explant model of a radial meniscus tear that a cell-seeded scaffold sheath could augment neotissue formation and associated mechanical properties through cell migration into the lesion and/or paracrine-mediated effects on the fibrochondrocytes in the native tissue (Shimomura et al. 2015). Nevertheless, the ex vivo culture conditions of our recent study did not replicate the mechanical demands or inflammatory mediators expected within an injured joint environment, tempering the generalization of these promising in vitro results to a clinical scenario. Stable integration of a scaffold sheath, mediated in part through enhanced suture retention strength, will be needed for translational success. Similarly, broad clinical adoption would likely require timely and secure fixation through an arthroscopic surgical approach. Related clinical studies have reported secure fixation of fascia sheaths (Henning et al. 1991) and collagen matrix membranes (Piontek et al. 2016) enveloping meniscus repairs, suggesting the feasibility of applying the scaffold sheet through an arthroscopic approach.

Beyond these opportunities to improve the scaffold design and implementation, there were several limitations inherent in this study. While the scaffold was fabricated to mimic the tensile properties of native menisci, its compressive and shear properties were not evaluated. In particular, a high coefficient of friction of the scaffold sheet could potentially abrade the articulating hyaline cartilage or enveloped meniscus. However, discrepancies between the friction coefficients of meniscus devices and native articular cartilage do not inevitably lead to joint degeneration, depending upon the ability of the device to support neotissue formation, including surface lubrication (Bonnevie et al. 2014; Lee et al. 2014). The immune response to the device is of similar concern. As PCL is a biodegradable, biocompatible biomaterial with a record of clinical safety (Sell et al. 2010), the scaffold sheet should not promote an adverse inflammatory response when implanted in vivo. Nevertheless, secure fixation of the scaffold to the meniscal lesion would be required to prevent dislodgement, with possible disruption of normal joint articulation. At present, these concerns can only be sufficiently evaluated in a large animal model.

An additional limitation to this study was that the mechanical properties of the surgical repair were only evaluated under tension. Although commonly employed in similar studies to evaluate suture techniques (Bhatia et al. 2015; Branch et al. 2015; Herbort et al. 2010), this protocol does not replicate how the meniscus functions in vivo. Instead, the native meniscus encounters a complex loading environment consisting of tensile, compressive, and shear forces, exerted dynamically in the context of other joint tissues such as cartilage, ligaments, and synovium. Analysis of surface contact stresses, as well as joint kinematics via robotic systems, would provide further insight into how novel repair techniques and biomaterials affect time-zero mechanics (Maher et al. 2011). Similarly, dynamic loading protocols simulating gait could provide information on the stability of biomaterials under more physiological conditions. To that end, such investigations should ideally be performed with human cadaveric samples, although homologous structure-function relationships of the meniscus exist across species (Proffen et al. 2012). Ultimately, long-term preclinical and clinical studies will be required to determine the potential benefit of promising results in vitro.

## Conclusions

This study showed that a novel biomimetic scaffold fabricated by electrospinning could be incorporated into the repair of a radial meniscus tear without compromising the tensile properties of the repair. Future research will explore methods to enhance suture retention strength. Additionally, the effect of seeding the scaffolds with adult stem cells to further improve long-term durability and integration will be examined. With further modification, the scaffold presented in this study may provide a potential approach to enhance healing of meniscus tears in patients.

## Abbreviations

CSA: Cross-sectional area
DMF: Dimethylformamide
MSC: Mesenchymal stem cell
PCL: Poly-ε-caprolactone
SEM: Scanning electron microscopy
THF: Tetrahydrofuran
